# Association of triglyceride glucose index with diabetic retinopathy in middle-aged and elderly ambulatory type 2 diabetic patients

**DOI:** 10.3389/fendo.2025.1442230

**Published:** 2025-04-01

**Authors:** Qiong Yao, Shang-quan Liu

**Affiliations:** Department of Endocrinology, The Third Affiliated Hospital of Anhui Medical University (The First People’s Hospital of Hefei), Hefei, China

**Keywords:** type 2 diabetes mellitus, triglyceride glucose index, diabetic retinopathy, middle-aged and elderly population, ambulatory patients

## Abstract

**Background:**

Diabetic retinopathy (DR) is a major contributor to vision loss and blindness among working-age adults worldwide. While the relationship between the triglyceride glucose (TyG) index and DR in hospitalized patients has been demonstrated, research on the correlation between the triglyceride glucose (TyG) index and DR in ambulatory type 2 diabetes mellitus (T2DM) patients is still relatively limited.

**Methods:**

A cohort of 398 middle-aged and elderly T2DM patients who underwent outpatient physical examinations at the First People’s Hospital of Hefei City between 2012 and 2017 were included in this study. They were categorized into either the nondiabetic retinopathy group (296 cases in the NDR group) or the diabetic retinopathy group (102 cases in the DR group) based on the results of dilated fundus examinations. One-way logistic regression and LR backward multifactorial logistic regression analyses were utilized to identify the risk factors associated with the development of diabetic retinopathy in T2DM patients. Furthermore, the predictive value of the TyG index for diabetic retinopathy in middle-aged and elderly ambulatory T2DM patients was evaluated through stratified regression to adjust for other factors, along with receiver operating characteristic (ROC) curve analysis.

**Results:**

Multivariate logistic regression analysis indicated that the TyG index was identified as an independent risk factor for diabetic retinopathy (DR) in middle-aged and elderly patients with type 2 diabetes mellitus (T2DM) (*P* < 0.05). The receiver operating characteristic (ROC) curve analysis revealed that the area under the curve (AUC) was 0.585 [95% CI (0.524, 0.646)] (*P* = 0.011), with an optimal cut-off value of 9.115, corresponding to a sensitivity of 82.4% and specificity of 36.1%.

**Conclusion:**

The TyG index serves as an independent risk factor for diabetic retinopathy (DR) in middle-aged and elderly ambulatory type 2 diabetes mellitus (T2DM) patients, and it is recommended that this index be used as a reference index in the comprehensive assessment of DR.

## 1 Introduction

Diabetic retinopathy (DR), a prominent diabetic microvascular complication, is notorious for inflicting severe retinal damage, leading to vision impairment and blindness. It stands as the primary cause of visual loss in individuals with diabetes and has emerged as a significant global public health concern (1). A comprehensive evaluation and meta-analysis (2)revealed an overall DR prevalence of 22.27% among diabetic patients, with vision-threatening DR (VTDR) affecting 6.17% of cases. By 2020, the global prevalence of DR among adults was estimated at approximately 103.12 million, with projections indicating continued growth in the future. Middle-aged and elderly individuals face elevated risk due to factors such as aging and prolonged disease duration. Moreover, beyond its high prevalence, DR is strongly linked to various ocular complications (e.g., cataracts, glaucoma) and systemic complications (e.g., cardiovascular disease, renal disease) (3, 4). Hence, there is a crucial need to investigate and address associated risk factors to enable early detection and intervention in the progression of DR.

Recent research has highlighted the strong association between insulin resistance (IR) and diabetic retinopathy (DR), suggesting its potential for assessing the severity of DR (5). While the hyperinsulinemic-normoglycemic clamp technique remains the “gold standard” for diagnosing IR, its clinical utility is limited by its invasive and costly nature. However, a recent global study has indicated that the triglyceride glucose (TyG) index offers a comparable alternative to the high insulin-normal glucose clamp technique for assessing insulin levels, demonstrating high sensitivity and specificity (6). Notably, the TyG index, derived from fasting triglycerides (TG) and fasting glucose (FPG), provides a simpler, more cost-effective, and reliable means of assessing IR compared to other indices, presenting a clear advantage in clinical practice.

In recent years, there has been a growing focus on the role of the TyG index in diabetes research. Several studies have highlighted its association with an elevated risk of macrovascular events and the development of microangiopathy in patients with type 2 diabetes mellitus (T2DM) (7). Additionally, an increased TyG index in hospitalized patients has been linked to a higher likelihood of developing diabetic vascular-related lesions (8). Furthermore, across-sectional observational study demonstrated a strong correlation between a higher TyG index and the presence of microangiopathy in diabetic patients, suggesting the potential use of the TyG index for monitoring individuals’ metabolic status in clinical settings (9).

However, the current research landscape predominantly focuses on hospitalized patients, with insufficient attention directed towards the general population of ambulatory patients. In contrast to hospitalized individuals, ambulatory diabetic patients often exhibit reluctance towards hospital admissions due to financial constraints, time constraints, and a lack of awareness regarding disease prevention, leading to low attendance rates. Consequently, early disease detection becomes challenging, resulting in delayed treatment interventions and subsequently heightened disability and mortality rates. Hence, there arises a critical need for a simple, reliable, and cost-effective screening tool to detect chronic complications in ambulatory patients promptly.

Therefore, through a retrospective analysis of clinical data from selected patients, this study aims to explore the correlation between the TyG index and diabetic retinopathy (DR) in middle-aged and elderly ambulatory patients with type 2 diabetes mellitus (T2DM). The overarching goal is to establish a foundation for screening and preventive strategies for this disease.

## 2 Subjects and methods

### 2.1 Study subjects

Three hundred and ninety-eight middle-aged and elderly patients diagnosed with type 2 diabetes mellitus (T2DM) who underwent outpatient physical examinations at the First People’s Hospital of Hefei City between 2012 and 2017 were included in this study. The inclusion criteria were as follows: (1) all patients met the diagnostic criteria for T2DM according to the American Diabetes Association (10); (2) were aged 40 years or older. Exclusion criteria included: (1) other types of diabetes mellitus; (2) recent acute complications of diabetes mellitus or acute infections; (3) incomplete data; and (4) coexisting malignant tumors, psychiatric disorders, or severe heart, liver, kidney, or other organ diseases. Based on the results of dilated fundus examinations, the selected patients were categorized into the non-diabetic retinopathy group (296 cases in the NDR group) and the diabetic retinopathy group (102 cases in the DR group).

The study was approved by the Ethics Committee of the First People’s Hospital of Hefei (Ethics Approval No. 2024-082-01).

### 2.2 Methods

In this study, a wide range of data was collected from the outpatient medical record system. This information included demographic details such as gender, age, diabetes history, and various biochemical measurements taken in the fasting state, including triglycerides (TG), total cholesterol (TC), low-density lipoproteins (LDL-C), high-density lipoproteins (HDL-C), blood creatinine (Cr), blood uric acid (UA), glycosylated hemoglobin (HbA1C), and fasting glucose (FPG).

### 2.3 Calculate the TyG index

TyG index was calculated according to the formula of TyG=ln [TG (mg/dl) x FPG (mg/dl)/2] (11).

### 2.4 Statistical methods

SPSS 25.0 statistical software and R version 4.2.2 were used for data analysis. Continuous variables conforming to normal distribution were expressed as mean ± standard deviation, and independent samples t-test was used for comparison between two groups; non-normally distributed data were expressed as median and interquartile range, and Mann-Whitney U-test was used for comparison between two groups; count data were expressed as relative numbers, and χ 2 test was used for comparison between groups. Univariate and LR backward multivariate logistic regression analyses were used to explore the factors influencing the occurrence of DR in middle-aged and elderly patients with ambulatory T2DM; the predictive value of TyG index for the occurrence of DR in elderly patients with T2DM was evaluated by applying the subject’s work characteristics (ROC) curves, and the critical value of TyG index was determined. The difference was considered statistically significant at *P*<0.05.

## 3 Result

### 3.1 General clinical data of the two groups

Following fundus examinations, the 398 middle-aged and elderly individuals with T2DM were stratified into the non-diabetic retinopathy group (NDR group, 296 cases) and the diabetic retinopathy group (DR Group, 102 cases). The results demonstrated significant discrepancies in age, smoking history, TG, UA, and TyG index between the two groups (*P*<0.05). Conversely, no notable variances were observed in the remaining observational indicators (*P*>0.05) (See **Table 1**).

**Table 1:** Comparison of clinical data of patients [n(%), x ± s].

Variables	NDR Group (n=296)	DR Group (n=102)	*P*
Age (year)	55.29 ± 12.10	58.43 ± 11.32	0.022^*^
Course (month)	60.00 (24.00,120.00)	72.00 (33.00,120.00)	0.205
BMI (kg/m^2^)	24.33 (22.66,26.64)	24.83 (22.74,26.84)	0.224
Systolic pressure (mmHg)	126.00 (120.00,130.00)	124.50 (118.00,134.25)	0.735
Diastolic pressure (mmHg)	80.00 (75.00,88.00)	80.00 (70.00,90.00)	0.982
TG (mmol/L)	1.75 (1.14,2.46)	2.03 (1.37,2.63)	0.013*
TC (mmol/L)	4.75 (4.1,5.50)	4.79 (4.15,5.71)	0.529
LDL-C (mmol/L)	2.65 (2.09,3.26)	2.56 (2.01,3.10)	0.169
HDL-C (mmol/L)	1.22 (1.02,1.50)	1.25 (1.08,1.62)	0.132
HbA1C (%)	8.20 (7.03,9.30)	8.20 (7.10,9.50)	0.551
FPG (mmol/L)	8.80 (7.10,10.80)	9.05 (7.48,11.33)	0.142
CR (umol/L)	61.85 (50.93,72.95)	63.65 (56.48,74.43)	0.082
UA (umol/L)	286.08 ± 76.64	309.42 ± 83.40	0.010^*^
TyG	9.44 (8.86,9.89)	9.56 (9.19,9.94)	0.011*
Gender [n (%)]			0.286
Male	159 (53.72%)	61 (59.80%)	
Female	137 (46.28%)	41 (40.20%)	
Smoking [n (%)]			0.048^*^
No	219 (73.99%)	65 (63.73%)	
Yes	77 (26.01%)	37 (36.27%)	
Drinking [n (%)]			0.293
No	264 (89.19%)	87 (85.29%)	
Yes	32 (10.81%)	15 (14.71%)	
Family history [n (%)]			0.141
No	198 (66.89%)	60 (58.82%)	
Yes	98 (33.11%)	42 (41.18%)	

TG, triglyceride; TC, total cholesterol; HDL-C, high-density lipoprotein cholesterol; LDL-C, low-density lipoprotein cholesterol; TyG, triglyceride glucose index; CR, creatinine; UA, uric acid; FPG, fasting plasma glucose; HbA1C, glycosylated hemoglobin. *P<0.05.

### 3.2 Univariate and multivariate logistic regression analyses

Upon accounting for variables such as gender, age, smoking history, and blood pressure, univariate logistic regression analysis was employed to evaluate the risk factors associated with diabetic retinopathy (DR) among hospitalized individuals. The findings indicated that age, smoking history, serum uric acid (UA), triglycerides (TG), and the TyG index were significant risk factors for DR in middle-aged and elderly patients with type 2 diabetes mellitus (T2DM). However, no notable differences were observed concerning other indicators. Subsequently, significant variables from the univariate analysis were included in a backward stepwise logistic regression analysis (LR) to control for confounding factors. Given that the calculation of the TyG index involves TG and fasting blood glucose (FPG), these two variables were excluded from the regression model to mitigate multicollinearity issues. Following adjustments for age, drinking history, and other relevant factors, the association between the TyG index and DR risk was examined. The results revealed that both the TyG index (OR=1.641, *P*=0.003, 95%CI=1.180-2.282) and age (OR=1.036, *P*=0.001, 95%CI=1.014-1.059) were independent risk factors for DR after controlling for confounding factors (See **Table 2**).

**Table 2:** Univariate and multivariate logistic regression analyses.

Variables	Univariate Logistic Regression		Multivariate Logistic Regression	
	OR (95%*CI*)	*P*	OR (95%*CI*)	*P*
Gender	0.780 (0.494, 1.232)	0.287		
Age	1.023 (1.003, 1.043)	0.023*	1.036 (1.014, 1.059)	0.001*
Course	1.002 (0.999, 1.005)	0.237		
BMI	1.044 (0.973, 1.122)	0.231		
Systolic pressure	1.001 (0.986, 1.017)	0.880		
Diastolic pressure	1.003 (0.980, 1.026)	0.821		
TG	1.132 (1.006, 1.274)	0.039*		
TC	1.049 (0.870, 1.266)	0.615		
LDL-C	0.840 (0.656, 1.077)	0.169		
HDL-C	1.571 (0.932, 2.648)	0.090		
HbA1C	1.062 (0.898, 1.256)	0.482		
FPG	1.051 (0.980, 1.127)	0.162		
CR	1.007 (0.997, 1.018)	0.161		
UA	1.004 (1.001, 1.007)	0.011*	1.003 (1.000, 1.006)	0.093
TyG	1.539 (1.135, 2.089)	0.006*	1.641 (1.180, 2.282)	0.003*
Smoking	1.619 (1.002, 2.616)	0.049*	1.643 (0.978, 2.760)	0.060
Drinking	1.422 (0.736, 2.751)	0.295		
Family history	1.414 (0.890, 2.246)	0.142		

TG, triglyceride; TC, total cholesterol; HDL-C, high-density lipoprotein cholesterol; LDL-C, low-density lipoprotein cholesterol; TyG, triglyceride glucose index; CR, creatinine; UA, uric acid; FPG, fasting plasma glucose; HbA1C, glycosylated hemoglobin. *P<0.05. (OR) value, odds ratio; (95% CI) 95% confidence interval; (ROC) curves, Receiver operating characteristic. *P<0.05.

### 3.3 Multivariate logistic analysis of TyG index quartile subgroup and DR

In order to establish the relationship between the TyG index and diabetic retinopathy (DR), all patients were categorized into four groups based on the quartiles of the TyG index (Q1: TyG ≤ 8.96, Q2: TyG 8.96-9.47, Q3: TyG 9.47-9.92, Q4: TyG > 9.92, as shown in **Table 3**). The odds ratio (OR) for DR in the highest quartile compared to the lowest quartile was 2.57 (95% CI 1.26-5.24, *P* = 0.009) (**Table 3**). Even after adjusting for sex, age, smoking history, triglycerides (TG), and serum uric acid (UA), the risk of DR remained significantly elevated in the highest quartile compared to the lowest quartile (OR 3.14, 95% CI 1.17-8.42, *P* = 0.023) (See **Table 3**).

**Table 3:** Multivariate logistic analysis of TyG index quartile subgroup and DR.

	Model 1		Model 2		Model 3	
	OR (95%*CI*)	*P*	OR (95%*CI*)	*P*	OR (95%*CI*)	*P*
Q1	1.000	–	1.000	–	1.000	–
Q2	2.45 (1.20, 5.00)	0.014*	2.94 (1.41, 6.14)	0.004*	2.85 (1.33, 6.09)	0.007*
Q3	2.83 (1.40, 5.74)	0.004*	3.24 (1.57, 6.69)	0.001*	3.66 (1.67, 7.99)	0.001*
Q4	2.57 (1.26, 5.24)	0.009*	3.50 (1.65, 7.45)	0.001*	3.14 (1.17, 8.42)	0.023*

Model 1: Non-adjusted; Model 2: Adjusted 1 for: gender, age; Model 3: Adjusted 1 for: gender, age, smoking history, TG, UA. Q1: TyG ≤ 8.96; Q2: TyG 8.96-9.47; Q3: TyG 9.47-9.92; Q4: TyG > 9.92; OR value, odds ratio; 95% CI, 95% confidence interval; ROC curves, Receiver operating characteristic. *P<0.05.

### 3.4 Hierarchical logistic regression analysis model

**Figure 1:**
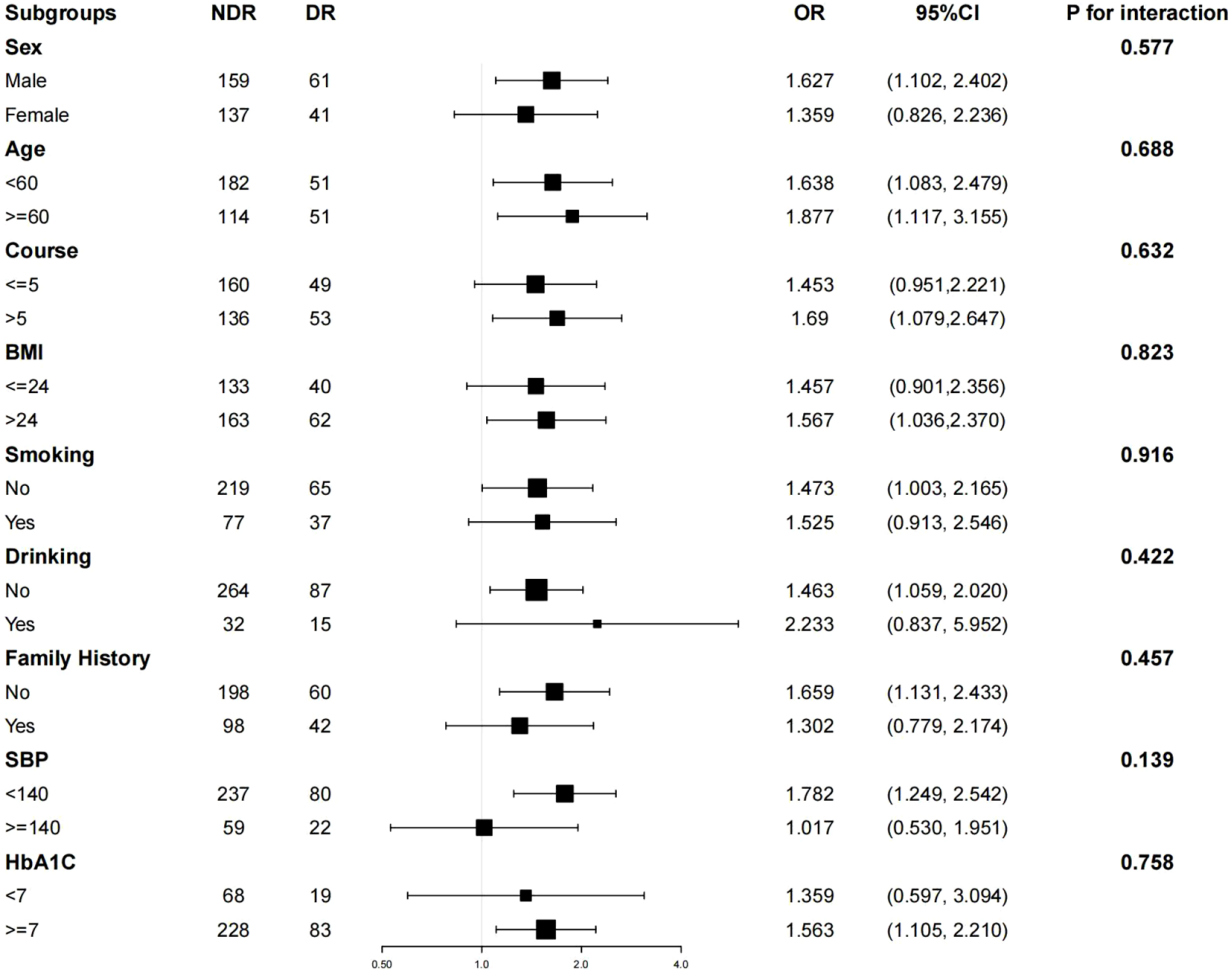
Hierarchical logistic regression analysis model to explore the variables affecting the correlation between TyG index and DR. The model was based on age (< 60 years vs >=60 years), sex (female vs male), duration of disease (<=5 years vs > 5 years), BMI (<=24 kg/m2 vs > 24 kg/m2), smoking history, drinking history, family history of diabetes mellitus, systolic blood pressure (< 140 mmHg vs>=140 mmHg) and HbA1C (< 7% vs >= 7%) were adjusted.

Subgroup analysis indicated that variables such as age (< 60 years vs. >= 60 years), sex (female vs. male), duration of disease (<= 5 years vs. > 5 years), BMI (<= 24 kg/m2 vs. > 24 kg/m2), smoking history, drinking history, family history of diabetes mellitus, systolic blood pressure (< 140 mmHg vs. >= 140 mmHg), and HbA1C (< 7% vs. >= 7%) exhibited no statistically significant impact on the interaction between the TyG index and DR-related effects (P > 0.05 for both interactions), as depicted in **Figure 1**. These results suggest that the various stratification factors in the model do not influence the correlation between the TyG index and DR (refer to **Figure 1**).

### 3.5 ROC curve analysis

**Figure 2:**
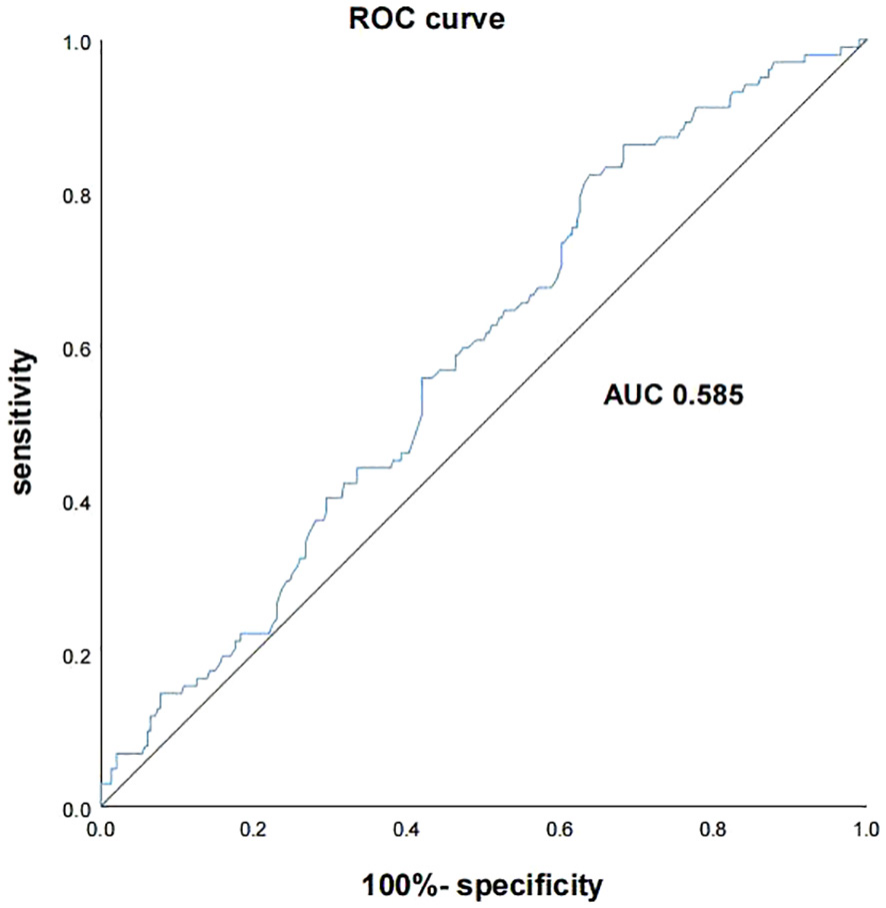
ROC curves of triglyceride glucose index predicting diabetic retinopathy.

An ROC curve analysis was conducted to evaluate the predictive ability of the TyG index for diabetic retinopathy (DR) in type 2 diabetes mellitus (T2DM) patients. The ROC curve utilized the TyG index as the test variable and DR (assigned = 1) as the state variable. The AUC was calculated to be 0.585 [95% CI (0.524, 0.646)] (*P* = 0.011), indicating fair predictive performance. The optimal diagnostic threshold was identified as 9.115, with corresponding sensitivity of 82.4% and specificity of 36.1% (refer to **Figure 2**).

## 4 Discussion

The study’s findings highlight the TyG index as an independent risk factor for diabetic retinopathy (DR) in patients with type 2 diabetes mellitus (T2DM), showcasing a strong correlation with the disease. This positions the TyG index as a promising, reliable indicator for future DR screening in middle-aged and elderly T2DM patients within community-based populations. Computed as the product of triglyceride and fasting glucose levels, the TyG index reflects disruptions in both glucose and fat metabolism, offering a dependable alternative biomarker for insulin resistance (IR) (12). Its versatility extends to predicting cardiovascular disease risk and prognosis, playing a crucial role in assessing cardiovascular disease, diabetes mellitus, and metabolic syndrome (13, 14). In recent years there has been a growing interest in research related to its association with diabetes and its complications.

DR is a prevalent microvascular complication of diabetes, affecting approximately 1 in 3 diabetic patients. The risk of developing DR increases significantly with the duration of the disease (15). In China, a lack of awareness about diabetes and its chronic complications (16), combined with atypical symptoms and signs during the early stages of the disease (17), often leads to patients seeking medical attention only after experiencing vision loss or even blindness. This delayed diagnosis results in a low recognition and diagnosis rate of DR, with many cases already at an advanced stage and carrying a poor prognosis.

Non-hospitalized patients, in contrast to their hospitalized counterparts, who receive timely formal examinations and health guidance, have fewer systematic opportunities for disease prevention and education, thus increasing their risk of developing chronic diseases with more severe progression. Therefore, our study emphasizes screening and prevention strategies for ambulatory patients, aiming to reduce the overall disease burden and minimize the risk of complications.

The relationship between the TyG index and the development of DR remains a subject of ongoing debate. While some studies have provided evidence supporting this association (9), a retrospective cross-sectional study conducted by Chiu et al. (18) did not find a clear correlation between DR and the TyG index. However, in our present study, we rigorously adjusted for various confounding factors using multiple models and uncovered a significant link between the TyG index and the incidence of DR in ambulatory patients with T2DM. Discrepancies in findings across studies may be attributed to factors such as the composition of the study population, inclusion and exclusion criteria, study design, and statistical methodologies. In light of these divergent outcomes, clinicians should be mindful to focus on monitoring this index when evaluating and managing patients with T2DM.

The development of DR is influenced by a myriad of factors, including gender, age, duration of diabetes, blood glucose levels, obesity, and smoking (19). Recent research has delved into the correlation between the TyG index and diabetic microvascular complications, shedding light on its potential significance (20). Notably, a study underscored the capability of an elevated TyG index in patients with T2DM to effectively identify high-risk groups necessitating early preventive interventions (21). Additionally, findings from a community-based survey revealed a compelling association between elevated TyG index levels and an augmented risk of arterial stiffness and renal microvascular injury in an elderly Chinese population, suggesting the potential utility of the TyG index in assessing vascular risk related to diabetic complications in outpatient settings (22). A meta-analysis conducted by Zhou et al. (23) suggested that a higher TyG index may be linked to an increased prevalence of DR in patients with type 2 diabetes mellitus. The above research indicates that there is a certain correlation between the TyG index and the occurrence of diabetes and its related complications.

The results of this study are consistent with them, indicating that TyG index is an independent risk factor for DR in T2DM combined. After adjusting for confounding factors such as gender, age, and smoking history, the risk of developing DR in the Q4 group with a TyG index is 3.14 times that of the Q1 group, indicating that a high TyG index is a risk factor for DR in middle-aged and elderly patients. Meanwhile, ROC curve analysis showed that the sensitivity of TyG index in predicting DR was 82.4%, which is highly sensitive and can help identify high-risk groups, and has high clinical significance for the early prevention, early diagnosis and treatment of DR. Combined with the characteristics of this index such as simplicity, economy, reliability, etc., this index may be widely promoted. However, the area under the ROC curve and the specificity were low in this study, which might be due to the small sample size, the susceptibility of the index itself to interference and the limitations of the selected population, which resulted in the bias of the data and underestimation of the occurrence of DR. Therefore, although the TyG index has some predictive value for the occurrence of DR, relying solely on the TyG index to predict DR is risky in clinical practice and may lead to more false-positive or false-negative results. In order to avoid these risks, it is recommended that the index be used as a reference index in the comprehensive assessment, combined with other clinical examinations and patient symptoms, to assist clinical workers in making a more comprehensive judgment.

The occurrence of DR varies significantly with its severity; however, relevant studies indicate that insulin resistance (IR) and the TyG index are associated with the occurrence and progression of DR as well as its severity. For instance, a cross-sectional study conducted by Irina et al. identified IR as an independent risk factor for diabetic proliferative retinopathy, demonstrating a significant correlation even after adjusting for multiple related variables (24). Furthermore, a hospital-based nested case-control study found a close relationship between the TyG index and different severities of DR (OR 0.83, 95% CI 0.72, 0.95; *P*=0.007), particularly with vision-threatening diabetic retinopathy (VTDR) (OR 0.53, 95% CI 0.36, 0.79; *P*=0.001), suggesting its potential as an early warning indicator for DR and VTDR (25). These research findings provide important evidence for our understanding of the onset and progression of DR, and also offer potential biomarkers and research directions for the early identification and intervention of DR and vision-threatening diabetic retinopathy (VTDR) in clinical settings. Subsequent studies can build upon this foundation to further explore the specific application value of the TyG index in the diagnosis and treatment of DR, as well as how to translate these findings into more effective clinical practices, thereby better assisting diabetic patients in preventing and controlling retinal diseases, and improving their quality of life and visual health.

The pathogenesis of DR is intricate, involving various components such as diabetic metabolic disorders, chronic low-grade inflammation, and oxidative stress (26). The precise mechanism by which the TyG index impacts DR remains unclear. Certain studies have suggested (23, 27)a positive correlation between the TyG index and inflammatory markers like leukocytes and C-reactive protein. Moreover, a high TyG index has been associated with endothelial dysfunction, inflammatory response, and oxidative stress. With the increase of TyG index, the release of various inflammatory factors increases, which leads to the damage of retinal capillary endothelium; at the same time, the process of leukocyte adhesion and aggregation is activated, which further exacerbates vascular occlusion, resulting in localized ischemia, and ultimately leading to impaired microcirculation in the retina. Another study delved deeper (28), indicating that the TyG index reflects the combined effects of “glycotoxicity” and “lipotoxicity”, and that glucose-lipid metabolism plays a key role in the pathogenesis of DR. On one hand (29, 30),the ability of glucose to cross the blood-retinal barrier (BRB) is a prerequisite for hyperglycemia-induced pathological changes in DR. The glucose transport process depends on the mediation of glucose transporter protein (GLUT) and sodium-glucose transporter protein (SGLT), and in diabetes mellitus (DM), the transport of glucose by these two proteins is increased, and there is an increased accumulation of glucose in the retinal cells, which is recycled through glycolysis and glycolytic collateral cycling of the uptake and metabolism of glucose by the retinal cells, which activates a variety of metabolic pathways, including the non-enzyme-dependent glycosylation, the protein kinase C pathway, oxidative stress, and the hexanedioic acid pathway, ultimately leading to microangiopathy, neurodegeneration, and low to moderate inflammation in the DR. On the other hand, disorders of lipid metabolism are also closely related to the development of DR (31).A combined lipidomic and metabolomic study showed that the expression levels of 85 lipids in the plasma of patients in the experimental group (DR group) were significantly different from those in the control group (NDR group), which may be related to the vulnerability of the retina to lipid peroxidation (32).The retina is rich in polyunsaturated fatty acids, and when oxidative stress and oxidative degradation of lipids occur, the wingless MMTV integration site (WNT) pathway, which plays a pathogenic role in DR, is activated, causing retinal inflammation and neovascularization. In addition, increased lipid peroxidation induced by free fatty acids leads to damage of retinal pigment epithelial (PRE) cells, which are critical for maintaining visual function, ensuring nutrient transport, and promoting the differentiation and survival of photoreceptors. Therefore, damage or dysfunction of PRE cells leads to a range of retinal pathologies, including monogenic retinal dystrophy, age-related macular degeneration (AMD), and retinal detachment (33).

Additionally, Kowluru (34) highlighted that in individuals with type 2 diabetes mellitus, the concomitant presence of elevated plasma lipids and high glucose accelerates mitochondrial damage, leading to aberrations in cellular metabolism and function. This, in turn, contributes to the loss of capillary cells and plays a role in the onset and progression of DR. Overall, elevated TyG index levels exert a detrimental effect on DR in patients with type 2 diabetes mellitus, consistent with our findings.

In conclusion, our findings underscore the TyG index as an independent risk factor for DR in the middle-aged and elderly ambulatory population with type 2 diabetes mellitus. Moreover, it is evident that a high TyG index poses a significant threat to patient health. Therefore, it is recommended that the TyG index be calculated at the time of the patient’s initial diagnosis of diabetes. This index is used as an early indicator of the patient’s risk stratification and is recorded along with other basic information (e.g., age, duration of disease, etc.). For example, in patients with a high TyG index, it can be initially determined that they may be at relatively high risk of developing DR. In addition, it can be combined with a routine eye examination. Consider the impact of the TyG index when performing routine examinations such as visual acuity examinations and intraocular pressure measurements. If the TyG index suggests a higher risk, the review interval can be shortened appropriately, even if there is no obvious abnormality in the routine eye examination for the time being. For fundus examinations, such as dilated fundus examination and fundus photography. When the TyG index is elevated, details of retinal microvascular changes, such as microangiomas, hemorrhages, and other lesion features, can be more carefully observed during fundus examinations, and a more accurate prediction model can be established using the TyG index in combination with the fundus examination results to help doctors determine the rate of lesion progression.

## 5 Limitation

The results of this study need to be interpreted in the context of its limitations. First, this was a cross-sectional study and causal extrapolation of the results is not possible. Second, the study was conducted only on T2DM patients with regional physical examinations and included a limited sample size, so caution should be exercised when extrapolating to other subjects. Third, there are many confounding variables that can affect the correlation between TyG index and DR risk, such as new markers like mean platelet volume (MPV) and platelet distribution width (PDW), and the above variables were not included in our study, which may have had some impact on the results. Therefore, further validation in larger and more diverse cohorts is needed to confirm our findings and improve the applicability of the TyG index in the clinical setting.

## Data Availability

The raw data supporting the conclusions of this article will be made available by the authors, without undue reservation.
